# Experimental stress during molt suggests the evolution of condition‐dependent and condition‐independent ornaments in the king penguin

**DOI:** 10.1002/ece3.3677

**Published:** 2017-12-20

**Authors:** Quentin Schull, Jean‐Patrice Robin, F. Stephen Dobson, Hédi Saadaoui, Vincent A. Viblanc, Pierre Bize

**Affiliations:** ^1^ Université de Strasbourg CNRS, IPHC Strasbourg France; ^2^ Department of Biological Sciences Auburn University Auburn AL USA; ^3^ Institute of Biological and Environmental Sciences University of Aberdeen Scotland, UK

**Keywords:** Corticosterone, honest signal, Immunity, sexual selection, social selection

## Abstract

Sexual selection and social selection are two important theories proposed for explaining the evolution of colorful ornamental traits in animals. Understanding signal honesty requires studying how environmental and physiological factors during development influence the showy nature of sexual and social ornaments. We experimentally manipulated physiological stress and immunity status during the molt in adult king penguins (*Aptenodytes patagonicus*), and studied the consequences of our treatments on colourful ornaments (yellow‐orange and UV beak spots and yellow‐orange auricular feather patches) known to be used in sexual and social contexts in this species. Whereas some ornamental features showed strong condition‐dependence (yellow auricular feather chroma, yellow and UV chroma of the beak), others were condition‐independent and remained highly correlated before and after the molt (auricular patch size and beak UV hue). Our study provides a rare examination of the links between ornament determinism and selection processes in the wild. We highlight the coexistence of ornaments costly to produce that may be honest signals used in mate choice, and ornaments for which honesty may be enforced by social mediation or rely on genetic constraints.

## 1 INTRODUCTION

Understanding the evolution of conspicuous ornaments, often costly to produce and maintain, has been a long‐standing focus of evolutionary biology (Andersson, 1994; Kuijper, Pen, & Weissing, 2012). Darwin (1871) laid the groundwork for this topic by observing that conspicuous ornaments could enhance access to sexual partners and reproduction, and that their evolution might be explained by sexual selection. In the second half of the twentieth century, researchers realized that ornaments could also be used in competition for nonsexual resources, such as access to food and territories outside reproduction (Tobias, Montgomerie, & Lyon, 2012; West‐Eberhard, 1983). West‐Eberhard (1983) pointed out in her theory of social selection that ornaments can evolve whenever they enhance gene replication due “to differential success in social competition, whatever the resource at stake” (West‐Eberhard, 1983). Consequently, sexual selection can be viewed as a “subset of social selection in which the resource at stake is mates” (Lyon & Montgomerie, 2012; West‐Eberhard, 1983). However, drawing the line between ornaments that are under sexual versus “nonsexual social” selection (hereafter “social selection”) can be complicated as many ornaments have multiple functions. For example, ornaments used for courtship behavior may also be used for year‐round territory defense (Tobias et al., 2012).

Regardless of function or selection process, ornament evolution may depend on how honestly they reflect the health, vigor, or dominance of their bearer (Tanaka, 1996; Zahavi, 1975). Three alternative mechanisms have been proposed to guarantee signal honesty: condition‐dependence, social mediation, or genetic constraint (Hill, 2014a). Whereas the condition‐dependent signaling hypothesis states that individuals in better condition can afford to produce ornaments of higher quality highlighting the cost of production and/or maintenance (Hill, 2011), the social mediation and genetic constraint hypotheses are frequently invoked to explain the occurrence of trait production that is condition‐independent (Hill & Brawner, 1998; Roulin, 2016). Under the social mediation hypothesis, the costs of bearing bright ornaments may be defrayed only after trait production, whereas the genetic constraint hypothesis implies that the ornament is not necessarily costly to produce, but it is nonetheless difficult or impossible to cheat (genetically linked) (Hamilton & Zuk, 1982).

We experimentally tested for condition‐dependence in ornament production in a monomorphic seabird, the king penguin (*Aptenodytes patagonicus*), by manipulating birds' physiological status during molt. King penguins are brightly colored seabirds that breed in large colonies (>10,000 breeding pairs) throughout the subAntarctic islands. They display showy ornaments with three different modalities of color production (Table 1). Orange beak spots contain both exogenous carotenoid pigments (McGraw et al., 2007) and specialized stacks of elongated lamellae (resulting from cellular specialization) that also reflect structural UV colors (Dresp & Langley, 2006). In addition, king penguins possess orange auricular feather ornaments that contain an endogenously synthetized pterin pigment (Thomas, McGoverin, McGraw, James, & Madden, 2013). Experimental reductions of beak UV reflectance and auricular patch size have been shown to decrease the likelihood of pairing and thus the initiation of a reproductive event (Jouventin, Nolan, Dobson, & Nicolaus, 2008; Nolan et al., 2010; Pincemy, Dobson, & Jouventin, 2009), demonstrating their important use in mutual mate choice. Correlative studies have also reported that individuals with larger auricular patches are more aggressive (Viera, Nolan, Côté, Jouventin, & Groscolas, 2008) enabling them to occupy more central breeding territories in the colony thought to be of greater reproductive value (Keddar, Jouventin, & Dobson, 2015; Viera et al., 2008). Those results suggest that auricular patch size may function as a social signal of dominance. Further, the color of yellow breast feathers (also containing the endogenously synthetized pterin pigment) has been related to innate immunity (Nolan, Dobson, Dresp, & Jouventin, 2006), suggesting that genes involved in the production of that specific pigment may also be linked to the immune system. There is also support for links between ornament colors and various condition indices, including body size and condition, stress status, and metabolic rate (Keddar, Couchoux, Jouventin, & Dobson, 2015; Schull et al., 2016; Viblanc et al., 2016) (see Table 1). Finally, the beak spot ornament is a dynamic signal, reflecting short‐ to medium‐term physiological changes in parasite loads and fasting status (Schull et al., 2016). However, experimental studies are now required to test whether trait production (i.e., beak spot and auricular patch coloration and size) is condition‐dependent. A positive association between indices of condition and social ornaments may be explained by social dominance and increased access to resources, rather than by a cost of ornament production itself (e.g., Gonzalez, Sorci, & De Lope, 1999).

**Table 1:** Summary table of the relationships found in previous studies between color ornament features and behavioral or physiological features in king penguin

Ornament	Origin	Color feature	Physiological/Behavioral feature	Relationship	Sex	References
Beak spot	Pigmentary (Yellow–Orange)	Chroma	Body condition	+	Female	Viblanc et al., 2016;
						Schull et al., 2016;
		
			Energetic reserves (fasting period)	+	Male	
			Ticks load	‐	Female & male	
	Structural color (UV)	Brightness	Delay in pairing	‐	Female & male	Nolan et al., 2010;
			Body condition	+	Male	Dobson et al. 2008;Viblanc et al., 2016;
				‐	Female	Viblanc et al., 2016;
			Resting heart rate	+	Female & male	
		Hue	Oxidative damages	‐	Male	
			% CORT increase after stress (capture)	‐	Female & male	
		Chroma	Delay in pairing	‐	Female & male	Nolan et al., 2010;
			Ticks load	+	Female & male	Schull et al., 2016;
Auricular patch	Morphometry	Size–area	Delay in pairing	‐	Female & male	Jouventin et al., 2008; Pincemy et al., 2009; Nolan et al., 2010;
			Aggressivity/more central position in the colony	+	Female & male	Viera et al., 2008; Keddar, Couchoux et al.,^2015^; Keddar, Jouventin et al.,^2015^;
			Natural antibodies level	‐	Female & male	Viblanc et al., 2016;
	Pigmentary (Yellow–Orange)	Brightness	Delay in pairing	+	Male	Pincemy et al., 2009;
		Chroma & hue		‐	Male	
Breast Patch	Pigmentary (Yellow–Orange)	Brightness	Delay in pairing	+	Male	Pincemy et al., 2009;
		Chroma & hue		‐	Male	
		Hue	Cellular immune response (PHA)	‐	Male	Nolan et al., 2006

**Figure 1:**
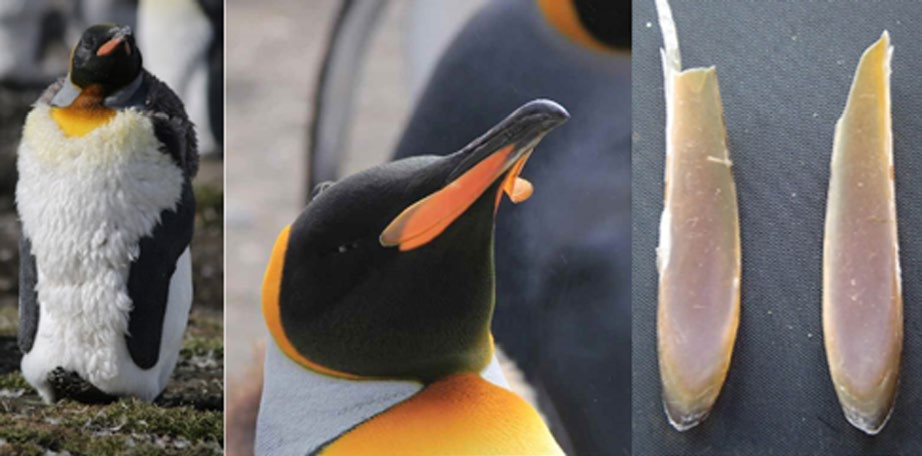
The molt of king penguins (*Aptenodytes patagonicus*). During the molt, king penguins renew their entire plumage (left panel) and their beak spot on each side of the beak (middle panel). The old keratin‐based beak spots are shed (right panel)

In the king penguin, both the entire plumage and the yellow–orange keratin beak plates are renewed each year during the molt, which occurs over a period of 3–4 weeks (Schull et al., 2016; see Figure 1). This particular context of a complete renewal (i.e., production) of all ornaments over a short period of time provides an ideal opportunity to investigate the costs of production of ornaments used in sexual and social contexts, and thus to examine whether sexual and social ornaments differ in their costs of production. To do so, we experimentally subjected molting birds to increased chronic stress (elevated glucocorticoid levels) or to an immune challenge (lipopolysaccharide LPS injection). Both chronic stress and immune stress are energy costly processes that can divert resource investment from the production of showy ornaments (Faivre, Grégoire, Préault, Cézilly, & Sorci, 2003; Folstad & Karter, 1992). Thus, we predicted that increasing stress through glucocorticoid manipulation or immune stimulation would hinder the ability of treated birds to invest in showy ornaments during the molt, when compared to control individuals.

## 2 MATERIAL AND METHODS

### 2.1 Study species and experimental procedures

Experiments were performed in the breeding colony of “La Baie du Marin” on Possession Island, Crozet Archipelago (46°26′S, 51°52′E, south Indian Ocean) over two consecutive field sessions: from November to February in 2014 and 2015. King penguins initiating their molt can be easily identified. They return before the start of their breeding cycle from a long foraging trip, weighing >16 kilos (for the usual weight of 13 kilos at the beginning of the breeding cycle) and molt in specific areas of the colony. All molting birds used in this study were captured in those specific areas and weighed on average (±*SD*) 16.98 ± 0.83 kg at the start of their molt.

In 2014, we subjected 30 molting birds (15 treated, 15 controls) to an experimental increase in baseline glucocorticoid levels. Treated birds were implanted with a subcutaneous corticosterone (CORT, G‐111) pellet in the middle of their back, just above the hipline. Control birds were implanted with a placebo (SHAM implants, C‐111, ©Innovative Research of America) (see Thierry, Ropert‐Coudert, and Raclot (2013) for a description of the methodology in Adélie penguins). Both implants were designed to diffuse over a 21‐day period. For CORT implants, this represented a 100 mg CORT release over 21‐days. Similar implants in the Adélie penguin have been shown to result in a 2.4 times increase in baseline CORT levels, thus staying within the natural range observed in penguins, but mimicking a late fasting stage in this species (Spée et al., 2011). Breeding Adélie penguins present similar circulating CORT levels as king penguins during the molt (Bourgeon, Viera, Raclot, & Groscolas, 2007).

In 2015, we subjected 30 birds (15 treated, 15 controls) to an experimental immune challenge. A lipopolysaccharide (LPS) injection inducing sickness symptoms, including fever, and leading to a physiological immune cascade response was used (Johnson, Curtis, Dantzer, & Kelley, 1993; Koutsos & Klasing, 2001; Xie, Rath, Huff, Huff, & Balog, 2000). The effects of LPS on the immune response are short‐lived (a few days; Adler, Peng, Peng, & Klasing, 2001; van de Crommenacker et al., 2010; Costantini, Greives, Hau, & Partecke, 2014), and we therefore insured birds were chronically challenged over the entire period of the molt (2–4 weeks; Cherel, Leloup, & Le Maho, 1988; Bourgeon et al., 2007). Treated birds were thus injected 4 times (once every 3 days) with 2 mg of lipopolysaccharide (LPS) diluted in 1 mL of physiological serum (LPS from *Escherichia coli* 0111:B4 © Sigma Aldrich). To avoid any risks of anaphylactic shock, the LPS dose injected (ca. 0.13 mg/kg) was 38 times lower than what is typically used in poultry studies (5 mg/kg; Cheng et al. 2004). Treated birds systematically showed signs of local inflammation (swelling) at the site of injection, confirming the treatment was effective. Control birds were injected with 1 mL physiological solution only (SHAM) and never showed signs of local swelling.

### 2.2 Morphometric and ornamental measures

In both years, we obtained measurements of body mass and color ornaments at the beginning and end of the experiment. Both experimental and control birds were initially measured at the start of the molt before providing the implants, and were measured a second time when caught after returning from their postmolt foraging trip of ca. 15 days (9–25 days) in order to court and breed.

When initially caught, birds were transported to a nearby dry shelter (within 10 m of the colony), and their body mass was measured to the nearest 2 g using an electronic scale. Flipper length (indices of structural size) was also measured to the nearest 1 mm using a solid metal ruler. We then regressed body mass on flipper size (*F*_*1,43*_ = 6.50, *p *=* *.013, *R* ^2^ = .07) and used the residuals as an index of body condition.

**Figure 2:**
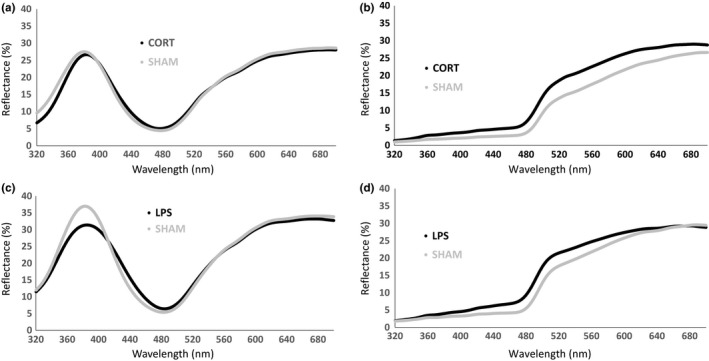
Comparison of average raw spectral data of adult king penguins (*Aptenodytes patagonicus*) at the end of their molt, depending on whether they were treated with a CORT implant, an LPS challenge, or respective SHAMs. (a) & (c) represent beak spectra, (b) & (d) auricular patch spectra. Each spectrum was averaged over all birds for each condition

Colors reflected by the beak spot and the auricular patches were measured using a portable JAZ spectrophotometer (Ocean Optics Inc., Dunedin, FL, USA) containing a pulsed‐xenon light with a spectral resolution of 0.3 nm across the spectral range of 320–700 nm, and was calibrated against a white standard (Ocean Optics Spectralon). Measures were repeated 3 times on each ornament (on both sides of the bird) using a 200 μm fiber‐optic probe with a 90° angle window. Reflectance spectra of given ornaments were smoothed and averaged using an R script adapted from Montgomerie (2008). The obtained spectra (e.g., see Figure 2) were used to calculate mean brightness, hue, and chroma (see below) over the spectral range 320–700 nm, which corresponds to the full range of spectral sensitivity in birds (Cuthill, 2006). King penguin beak spots show a reflectance peak in UV violet (320–490 nm) and a plateau in the yellow–orange portion (491–700 nm) of the spectrum (Schull et al., 2016), and we calculated color variables separately over those two regions. In contrast, feathered auricular patches contain a pterin‐based pigment (Thomas et al., 2013), only reflective above 450 nm. The spectral intensity, mean brightness (UV_brightness_ and YO_brightness_), was calculated by averaging reflectance over wavelengths: 320–490 nm and 491–700 nm for the beak, and 450‐700 nm for the auricular patch (Montgomerie, 2006). Hue is a measure of color appearance (e.g., “blue” and “yellow”). For the yellow–orange plateau portion of the spectrum, YO_hue_ was calculated as the wavelength at which the reflectance was halfway between its maximum and minimum (Keddar, Andris, Bonadonna, & Dobson, 2013). For the UV violet color of the beakspot, UV_hue_ was calculated as the wavelength of maximum reflectance between 320 and 490 nm. Finally, chroma is a measure of color purity and was calculated within the region of interest (UV_chroma_ and YO_chroma_) as the difference between maximum and minimum reflectance over the mean reflectance for that particular region (formula S8; Hill & McGraw, 2006, p. 108). In the king penguin, correlations between beak UV color parameters based on a large sample in previous experiments show that brightness, hue and chroma signal different information to breeding birds (for a discussion, see Schull et al., 2016 and ESM 1 therein). In contrast, yellow–orange color parameters are highly correlated both in the beak and in the ear feather patches (see Schull et al., 2016 and ESM 1 therein). We thus chose to focus on YO_chroma_ for both beak and auricular patch analyses, as this measure was the one presenting the highest among individual variation (thus containing the most information; (Dale, 2006), and directly reflects ornament pigment concentrations in several bird species (McGraw & Gregory, 2004; Saks, McGraw, & Hõrak, 2003).

### 2.3 Statistical analyses

All analyses were run in the statistical computing software R (v. 3.1.1; R development Core Team 2008). Differences between treated and control groups at the beginning of the molt and when returning from the postmolt foraging trip for breeding were investigated using linear models (LMs). We succeeded in recapturing 10 CORT and 8 control birds in 2014, and 13 LPS and 13 control birds in 2015, explaining the slight variation in sample sizes in our various analyses. No individual (identified by a radio frequency PIT‐Tag) was used repeatedly over the years. Bird sex (estimated during the molt based on a slight sexual dimorphism between males and females) could only be ascertained posteriorly during courtship when in direct morphometric comparison with the partner and using courtship songs (Jouventin, 1982; Jouventin & Dobson, 2017). Whereas only males were used in the LPS experiment (2015), 2 of the 10 CORT individuals and 2 of the 8 control birds in 2014 were females. Preliminary analyses showed no significant effects of sex, alone (LMs; 0.012 < *F*_*1,12*_ < 2.416, 0.142 < *p *<* *.914) or in interaction (LMs; 0.078 < *F*_*3,14*_ < 2.873, 0.114 < *p *<* *.785) with the CORT treatment; thus, sex was not retained in the final analyses. Running analyses with or without the females led to similar results, but decreased the power of our analyses. Therefore, it appeared reasonable to pool both sexes in our analyses. We found no evidence of a bias in size or coloration before the molt between individuals that were subsequently recaptured or not when returning from the postmolt foraging trip (LM models testing for effect of recapture [yes/no] alone or in interaction with treatment: 0.003 < *F*_*1,17*_ & *F*_3,14_ < 2.304; 0.139 < *p *<* *.955). Initial body condition at molt onset was treated as a covariate in the analyses to account for initial condition effects on color parameters. For analyses on the size of the auricular patch, flipper size (as a size proxy) was also controlled for as a covariate in the analyses. Relationships between body condition and color parameters were investigated using linear models with body condition as the response variable and all ornamental features as fixed factors. Nonsignificant terms were excluded following a backward stepwise procedure (Quinn & Keough, 2002). Effect sizes and 95% CI were calculated after Nakagawa & Cuthill (2007) for color variables measured at (A) molt initiation and (B) after the molt. Correlations between color variables (hue, chroma, and brightness) before and after molt were investigated using Pearson correlation tests. *F*‐statistics for fixed effects (tests of significant differences from zero) and *p*‐values are given. Effects were considered significant for *p *<* *.05. Residuals were visually inspected for normality using *qqplots* (opposing theoretical quantiles to sample quantiles).

### 2.4 Ethical statement

All experiments were approved by an independent ethics committee (Comités d'éthique Midi‐Pyrénées et Alsace pour l'expérimentation animale) and comply with the current laws of France. Authorizations to enter the breeding colony and handle the birds were provided by the “Terres Australes et Antarctiques Françaises” (permit n°2014‐127 issued on the 15 October 2014 and APAFIS#375 issued on the 17 July 2015).

## 3 RESULTS

### 3.1 Experiment 1: Corticosterone manipulation

**Figure 3:**
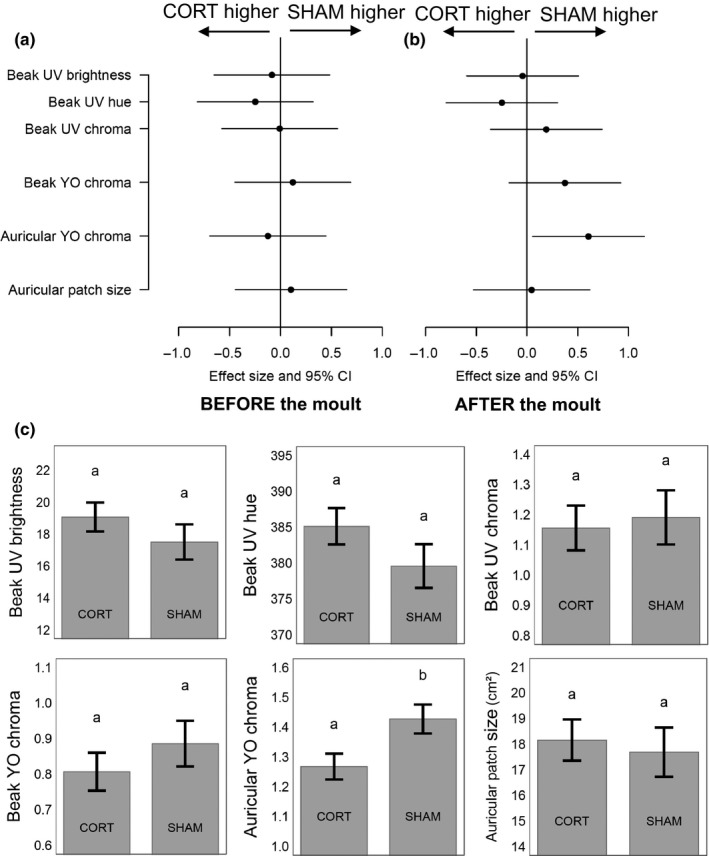
Pre‐ (panel a) and post‐ (panel b) molt comparison of beak and auricular patch color variables (controlled for body condition at the beginning of the molt), and auricular patch surface (controlled for structural size and body condition) in king penguins (*Aptenodytes patagonicus*) treated at molt initiation with a corticosterone (CORT) or sham implant. Panel c represents marginal means (±SE) of color variables measured after the molt

**Figure 4:**
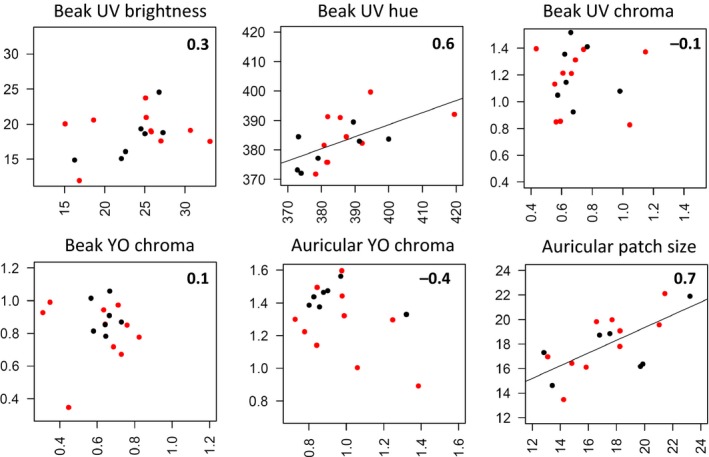
Correlation of beak and auricular patch color variables, and auricular patch surface before (*x*‐axis) and after the molt (*y*‐axis), for the same king penguins (*Aptenodytes patagonicus*). Pearson correlation coefficients are given in the top right corner. CORT‐ and sham‐treated birds are highlighted with red and black dots, respectively. A regression line is presented when the association is significant

At molt onset, CORT‐treated and control birds did not differ significantly in their ornamental features (LMs; treatment: 0.02 < *F*_*1,16*_ < 0.93, 0.341 < *p *<* *.964; Figure 3a) or body condition (LM; *F*_*1,16*_ = 0.02, *p *=* *.888). When returning from their postmolt foraging trip to court, birds treated with a CORT implant had significantly lower YO_chroma_ of their auricular patches (LM; *F*_*1,16*_ = 5.48; *p *=* *.033) (Figure 3b,c). Effects of the CORT treatment on beak and auricular color spectra are provided in Figure 2a,b. The CORT treatment had no effect on body condition (LM; *F*_*1,16*_ = 1.31, *p *=* *.271), beak spot coloration (LM; 0.066 < *F*_*1,16*_ < 1.812, 0.198 < *p *<* *.801; Figure 2a), and auricular patch size (LM; *F*_*1,15*_ = 0.12; *p *=* *.736; Figure 3b,c). The correlation between beak UV_hue_ before and after the molt was high (*r *=* *.59, *t *=* *2.85, *p *=* *.012, *N* = 18), as was that of auricular patch size (*r *=* *.67, *t *=* *3.53, *p *=* *.003, *N* = 18); other relations were nonsignificant (0.099 < *p *<* *.997, *N* = 18) (Figure 4).

Initial body condition was only related to the YO_chroma_ (estimate = 7.33 ± 1.88; *F*_*1,16*_ = 28.93, *p *<* *.001) and size (estimate = 0.391 ± 0.113; *F *=* *14.37, *p *=* *.002) of the auricular patch. No significant links were found between body condition at birds' return from their postmolt foraging trip and ornamentalal features (LM; 0.01 < *F*_*1,16*_ < 1.92, 0.199 < *p *<* *.979).

### 3.2 Experiment 2: LPS immune challenge

**Figure 5:**
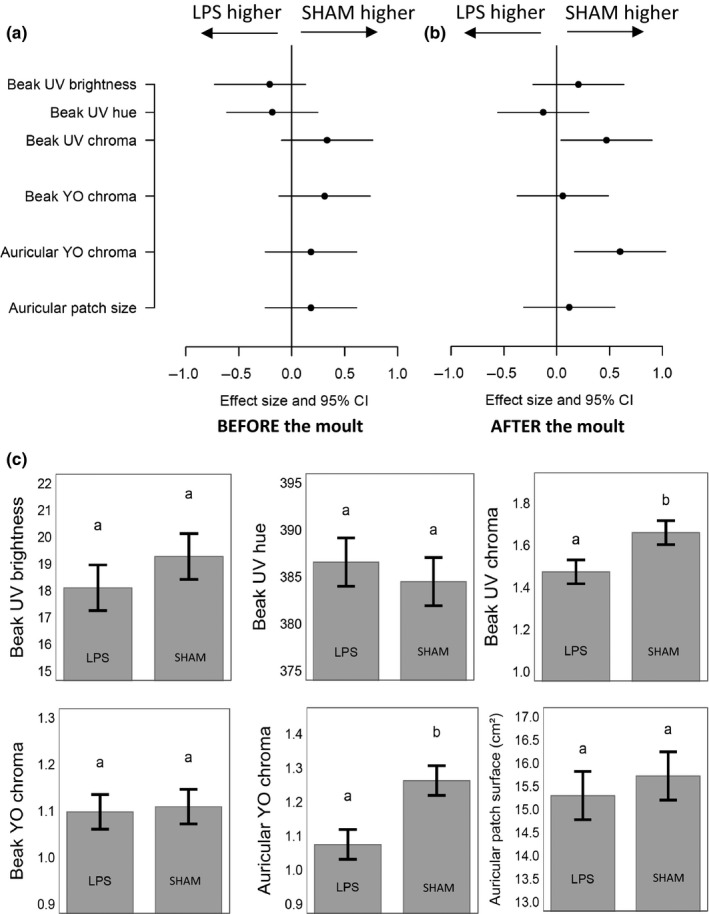
Pre‐ (panel a) and post‐ (panel b) molt comparison of beak and auricular patch color variables (controlled for body condition at the beginning of the molt), and auricular patch surface (controlled for structural size and body condition) for king penguins (*Aptenodytes patagonicus*) treated at molt initiation with lipopolysaccharide (LPS) or physiological serum (sham). Panel c represents marginal means (±SE) of color variables measured after the molt

**Figure 6:**
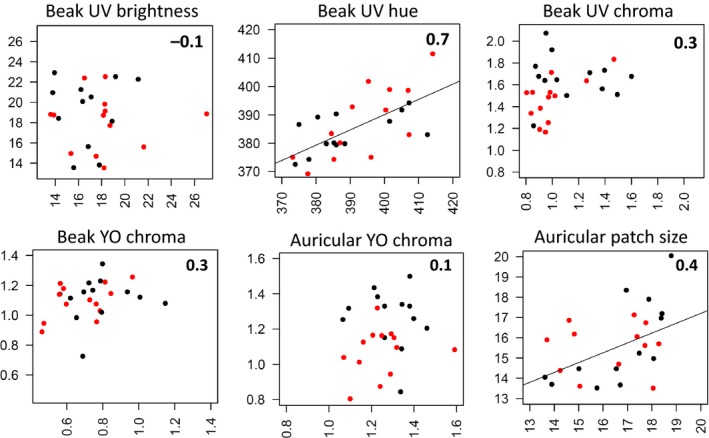
Correlation of beak, auricular patch color variables, and auricular patch size before (*x*‐axis) and after the molt (*y*‐axis), for the same king penguins (*Aptenodytes patagonicus*). Pearson correlation coefficients are given in the top right corner. LPS‐ and sham‐treated birds are highlighted with red and black dots, respectively. A regression line is presented when the association is significant

At molt onset, both treated (LPS) and control birds were similar in terms of ornamental features (LMs; treatment: 0.89 < *F*_*1,24*_ < 2.67, 0.120 < *p *<* *.360, Figure 5a) and body condition (LM; *F*_*1,24*_ = 0.45, *p *=* *.509). When returning from their postmolt foraging trip to court, both beak UV_chroma_ and the YO_chroma_ of the auricular patches were lower in LPS‐treated birds than in control birds (LMs; *F*_*1,24*_ = 5.98; *p *=* *.023 and *F*_*1,24*_ = 9.60; *p *=* *.005, respectively; Figure 5b,c). The effects of LPS treatment on beak and auricular color spectra are provided in Figure 2c,d. The LPS treatment had no significant effect on body condition (LM; *F*_*1,24*_ = 0.642, *p *=* *.432), on the other color parameters, and on the size of the auricular patches (LMs; treatment: 0.11 < *F*_*1,24*_ & *F*_*1,23*_ **< 1.02, 0.324 < *p *<* *.743; Figure 5b,c). Here again, the correlations for beak UV hue and auricular patch size measured before and after the molt were high (*r *=* *.65, *t *=* *4.25, *p *<* *.001, *N* = 26 and *r *=* *.44, *t *=* *2.41, *p *=* *.024, *N* = 26, respectively); other relations were non‐significant (0.123 < *p *<* *.698, *N* = 26) (Figure 6).

Initial body condition was related to auricular patch YO_chroma_ (LM; *F*_*1,24*_ = 4.67, *p *=* *.041). However, other parameters including the previous significant relation with auricular patch size (see Experiment 1) were not significantly related to initial body condition (LMs; 0.26 < F_*1,24*_ < 4.67; 0.157 < *p* < .609). No significant links were found between body condition at birds' return from their postmolt foraging trip and ornamental features (LM; 0.12 < *F*_*1,24*_ & *F*_*1,23*_ **< 1.37, 0.257 < *p *<* *.738).

## 4 DISCUSSION

Ornaments can be used in sexual and/or social interactions (Hill, 2014a; West‐Eberhard, 1983) and, regardless of the selection process, are expected to honestly reflect individual health, vigor, or social status in competitive contexts (Tanaka, 1996; Zahavi, 1975). Ornament honesty can be enforced by production costs (condition‐dependent signaling hypothesis; Hill, 2011), by the costs of bearing them in a social group (social mediation hypothesis; Rohwer, 1977), or by genetical constraints (i.e., linked to genes coding for immunocompetence; Whittingham, Freeman‐Gallant, Taff, & Dunn, 2015). Here, we investigated ornament production costs in adult king penguins by manipulating physiological stress and immunity status during the molt. Our results showed that some ornamental features were strongly affected by our treatments (auricular patch YO_chroma_ and beak spot UV_chroma_), while we found no evidence of treatment effects on other ornamental features (auricular patch size and beak spot UV_hue_). This demonstrates the coexistence of condition‐dependent and condition‐independent ornaments in adult king penguins.

### 4.1 Condition‐dependent ornaments

The condition‐dependent signaling hypothesis points out that the honesty of ornaments can come from unavoidable physiological or developmental costs of production (thus, preventing dishonest signals; Hill, 2011). This hypothesis predicts the existence of a trade‐off in energy or resource allocation between ornamentation and self‐maintenance. Thus, energetically challenging birds during ornament production (i.e., during the molt) by increasing the energy demand into self‐maintenance is expected to highlight trade‐off investments between those processes. Body condition of birds at the beginning of the molt was positively linked to the YO_chroma_ of their auricular patches in both experiments, suggesting that color production of that ornament is under an energetic trade‐off. Accordingly, ornaments that changed in response to CORT and LPS treatments showed a decrease in the purity of ornamental colors. Birds treated with CORT molted new auricular patches for which feather YO_chroma_ was lower than controls. Similarly, individuals repeatedly injected with LPS molted beak spots and ear feather patches for which UV_chroma_ and YO_chroma_, respectively, were both lower than in controls.

The so‐called stress hormone CORT is under the regulation of the hypothalamic‐pituitary‐adrenal (i.e., HPA) axis, and its release modulates energy allocation, allowing individuals to overcome challenging environmental conditions (Angelier, Wingfield, Weimerskirch, & Chastel, 2010). At short time scales, CORT release induces a rapid mobilization of energy resources, preparing the organism to cope with challenging situations (Peckett, Wright, & Riddell, 2011). Chronically elevated CORT leads to a reallocation of energy reserves into survival rather than other functions (Bókony et al., 2009; Spée et al., 2010), for example, by inhibiting vocalizations (Macdougall‐Shackleton et al., 2009), coloration (Roulin et al., 2008), courtship (Moore & Miller, 1984), and breeding behavior (Angelier & Chastel, 2009). In this study, penguins experimentally treated with a CORT implant may have invested less in the endogenous synthesis of pterins, resulting in a decreased allocation of this pigment to their ornaments reflected by lower YO_chroma_ of auricular patch feathers. Although changes in YO_chroma_ have been directly linked to carotenoid pigment concentrations in feathers in other bird species (McGraw & Gregory, 2004; Saks et al., 2003), yellow–orange coloration in the auricular patch of the king penguin (Thomas et al., 2013) is due to pterins (and not carotenoids). However, the costs associated with pterin endogenous synthesis and allocation remain unclear. Because pterins have been proposed to have antioxidant or immune functions (McGraw, 2005; Oettl, Greilberger, & Reibnegger, 2004; Weiss, Kennedy, Safran, & McGraw, 2011), those pigments could have been devoted to protection against CORT‐induced oxidative stress (Costantini, Marasco, & Møller, 2011) at the expense of auricular patch coloration.

The immunocompetence handicap hypothesis (a subhypothesis of the condition‐dependent signaling hypothesis) proposes that the production of ornaments comes at the expense of resistance to disease and parasites (Folstad & Karter, 1992). Several studies have shown positive links between immune efficiency and ornaments, notably ornamental coloration relying on exogenous pigment availability (Blount, Metcalfe, Birkhead, & Surai, 2003; Faivre, Préault et al., 2003), and reported a decrease in pigment‐based coloration in immune‐challenged individuals (Faivre, Grégoire et al., 2003). Thus, a trade‐off in the allocation of pigments toward colorful ornaments or immune functions may explain the honesty of some ornamental features. In this study, LPS treatment stimulating the immune function of molting king penguins led to decreased purity in the yellow–orange color of auricular patch feathers (lower YO_chroma_), which may result from the allocation of pterins to immune functions at the expense of auricular patch coloration. The LPS treatment also induced a decrease in purity of the UV coloration of the beak spot (UV_chroma_). Conversely to YO_chroma_ coloration of the auricular patch feathers, that is, due to pigments, beak spot UV reflectance in the king penguin has a structural basis resulting from crystal‐like photonic microstructures in the horny layer of the beak (Dresp & Langley, 2006). Our results suggest that the production of that photonic structure may be costly, especially in response to an immune challenge.

A previous study where king penguins were treated with an antiparasitic solution outside the molting period (i.e., during breeding) showed a strong increase in beak spot UV hue and brightness and a weak decrease in UV chroma after parasite removal (Schull et al., 2016). Although those results support a link between beak spot UV coloration, immunity and parasitism, we would have expected an increase in UV chroma in response to parasite removal and/or a decrease in UV hue and brightness in response to LPS treatments (Griggio, Zanollo, & Hoi, 2010; Leclaire, Pauline, Chatelain, & Gasparini, 2014; Schull et al., 2016). Those results call for a deeper understanding of the physiological and structural mechanisms leading to changes in UV hue, brightness, and chroma, notably the importance of production and maturation of photonic structures during the molt versus maintenance and modulation of those structures afterwards (e.g., during breeding).

### 4.2 Condition‐independent ornaments

Previous studies have shown that environmental factors encountered during ornament production (parasites, food shortage, or other stressors) do not necessarily strongly impact their expression (Hill & Brawner, 1998). In other words, the production/expression of those ornaments may be viewed as condition‐independent, raising questions about what factors enforce their honesty (Hill, 2013; Roulin, 2016). In contrast to auricular patch YO chroma, auricular patch size at the beginning of the molt was not consistently associated with birds' body condition (see differences between Experiments 1 and 2). One explanation could be that auricular patch size depends on the birds' condition at the time they are molted (i.e., birds' energy status during their production the previous year) but do not necessarily reflect the birds' condition 1 year later. However, the lack of association between ornamental features and condition at their return from the postmolt foraging trip in both experiments, suggests this is unlikely. Alternatively, a lack of consistent association suggests that auricular patch size may not be condition‐dependent. Indeed, we found no evidence that auricular patch size and beak spot UV hue were affected by CORT and LPS treatments. One hypothesis is that the honesty of condition‐independent ornaments is enforced by social mediation (Rohwer, 1977; West‐Eberhard, 1983). This hypothesis predicts that such ornaments should mirror social status and be constantly assessed during competitive interactions (Rohwer, 1977). Accordingly, auricular patch size in king penguins has been previously suggested to act as a social status badge, signaling individual competitiveness (Keddar, Couchoux et al., 2015; Viera et al., 2008). Individual penguins bearing larger auricular patches appear to be more aggressive and defend central breeding areas (Keddar, Couchoux et al., 2015; Viera et al., 2008) thought to be of higher quality (Bried & Jouventin, 2008) but see (Viblanc, Gineste, Stier, Robin, & Groscolas, 2014). The role of beak UV hue in social or sexual interactions is unknown. Experimental manipulation of auricular patch size (or beak UV hue) is now required to demonstrate whether bearing large auricular patches (or high UV wavelengths on the beak) entails socially mediated costs.

An alternative hypothesis for the evolution of condition‐independent ornaments in response to environmental challenges is that ornaments reflect the genetic quality of their bearer (i.e., genetical constraint hypothesis; Hill, 2014a). Indeed auricular patch size and beak spot UV hue were not affected by CORT and LPS treatments. Moreover, both traits showed similar expression before and after the molt. A similar strong relationship before and after the molt for beak spot UV hue was already observed in different individuals (Schull et al., 2016). Together, those findings indicate that the expression of auricular patch size and beak spot UV hue is maintained over time, which in turn suggests that their expression is largely determined by genetic or developmental factors encountered early in life that have lifelong consequences (e.g., silver spoon effects; Minias, Włodarczyk, Surmacki, & Iciek, 2015). However, the mechanisms linking individual genetic and epigenetic variation to trait production remain elusive. It has been recently proposed that ornament production could be constrained by the efficiency of vital cellular processes, with genes encoding for cellular metabolic pathways playing a key role in enforcing signal honesty (Hill, 2011, 2014a). Because the mitochondrion is the powerhouse of the cell, variation in mitochondrial and nuclear genes, epigenetic status, and mitonuclear interactions could account for interindividual genetic variation in ornament expression (Hill, 2014a; Hill & Johnson, 2013). Work using larger sample sizes, individuals of both sexes, and investigating mitochondrial function during and outside the period of ornament production may provide insightful information on the honesty of UV signals in the king penguin, and more generally on the importance of mitochondrial efficiency as a mediator of signal honesty in the animal kingdom (Hill, 2011, 2014a; Johnson & Hill, 2013).

## 5 CONCLUSION

Taken together, our study provides evidence for the evolution of condition‐dependent and condition‐independent ornaments in the king penguin, and suggests that variation in the cost of ornament expression could be rooted in their main use for mate attraction or for signaling social status. The expression of condition‐dependent ornaments was revealed by the decrease in chroma of the yellow–orange auricular feathers, most likely explained by a low deposition of pterin pigments in those feathers. Despite pterins being endogenously synthetized pigments, this finding suggests the evolution of a trade‐off in the allocation of pterin pigments to stress responses, such as antioxidant and immune functions, at the expense of colorful ornaments. This study also highlight that a unique ornament may evolve under non‐mutually exclusive selective forces.

## CONFLICT OF INTEREST

None declared.

## AUTHOR CONTRIBUTION

QS, VAV, and PB designed the study. QS, JPR, VAV, FSD, and HS did the fieldwork. QS, PB, and VAV analyzed the data and wrote the paper. FSD and JPR provided critical comments on the manuscript.
